# Case report demonstrating effectiveness of sorafenib in multiple lung and bone metastases of renal cell carcinoma

**DOI:** 10.3892/ol.2015.2844

**Published:** 2015-01-05

**Authors:** MANABU HOSHI, NAOTO OEBISU, JUN TAKADA, TDASHI IWAI, HIROAKI NAKAMURA

**Affiliations:** Department of Orthopedic Surgery, Osaka City University Graduate School of Medicine, Osaka, Japan

**Keywords:** renal cell cancer, sorafenib, bone metastasis, lung metastasis

## Abstract

The current study presents the case of a 59-year-old male with advanced-stage renal cell carcinoma and bone metastases in the proximal femur and ilium (cT3aN3M1; stage IV). Resection of the primary renal cell cancer and palliative surgery with a γ-nail for an impending fracture of the right proximal femur were performed, followed by radiotherapy. Sorafenib, a multi-kinase inhibitor that blocks the raf and tyrosine kinases of the vascular endothelial and platelet-derived growth factor receptors, was administered for 9 months, resulting in a marked improvement in the metastatic ilium and a reduction in the extent of the lung metastases. The patient suffered minor adverse effects, including a skin rash and mild diarrhea, but remained alive at the time of follow-up at 36 months post-surgery. Sorafenib demonstrated efficacy against the bone metastasis of metastatic renal cell carcinoma.

## Introduction

Renal cell carcinoma (RCC) accounts for ~3.8% of all adult malignancies in the USA (1). Recently, the number of patients diagnosed with RCC has increased due to the development of diagnostic modalities, including ultrasonography and CT (computed tomography). Approximately 20–30% of RCC patients develop bone metastases (2), which represents the third most common site of distant metastases in advanced RCC, following the lungs and liver (3).

Radiographically, bone metastases from RCC are predominantly osteolytic in nature and decrease bone integrity, leading to significant patient morbidity due to the associated skeletal-related events (SREs). SREs are defined as pathological fractures, radiotherapy for bone pain, surgery for impending fracture, spinal cord and nerve root compressions and hypercalcemia. SREs may significantly decrease patient quality of life. Radiotherapy is the most common SRE in RCC patients, as ~81% of patients with RCC receive radiotherapy treatment and 29% require orthopedic surgery (2).

Sorafenib is an epidermal growth factor receptor (EGFR) tyrosine kinase inhibitor that has been used in molecularly targeted therapy for advanced-stage renal cell cancer (RCC). In a study by Escudier *et al* (4), sorafenib treatment prolonged the median progression-free survival of RCC patients (5.5 months), when compared with a placebo group (2.8 months). However, there is little available information on the radiological effects of sorafenib on the bone metastases of RCC. The current study presents the case of a 59-year-old male with metastatic RCC and multiple metastases of the femur and ilium, who demonstrated marked recovery of the bone metastasis and reduction of the lung metastases, following treatment with sorafenib. Written informed consent was obtained from the patient.

## Case report

**Figure 1:**
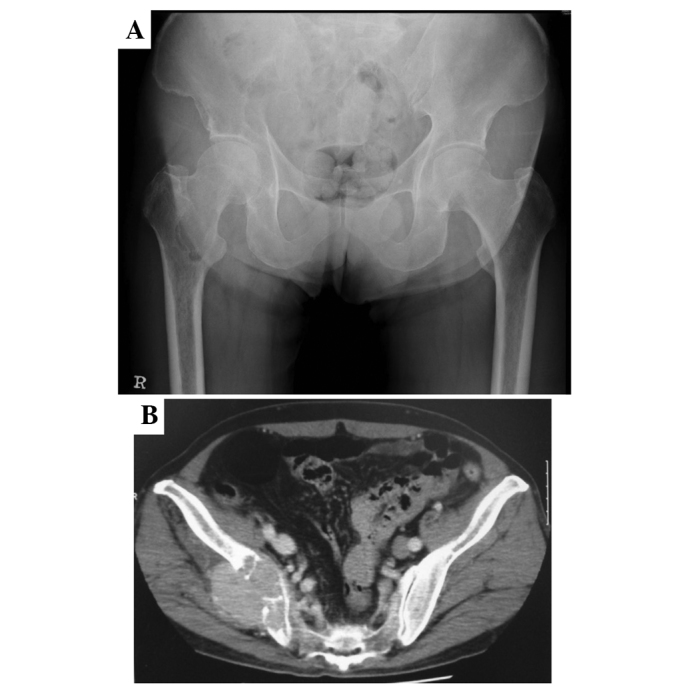
(A) Plain film showing an osteolytic lesion in the right proximal femur. (B) Pelvic computed tomography of a tumor lesion in the right ilium.

**Figure 2:**
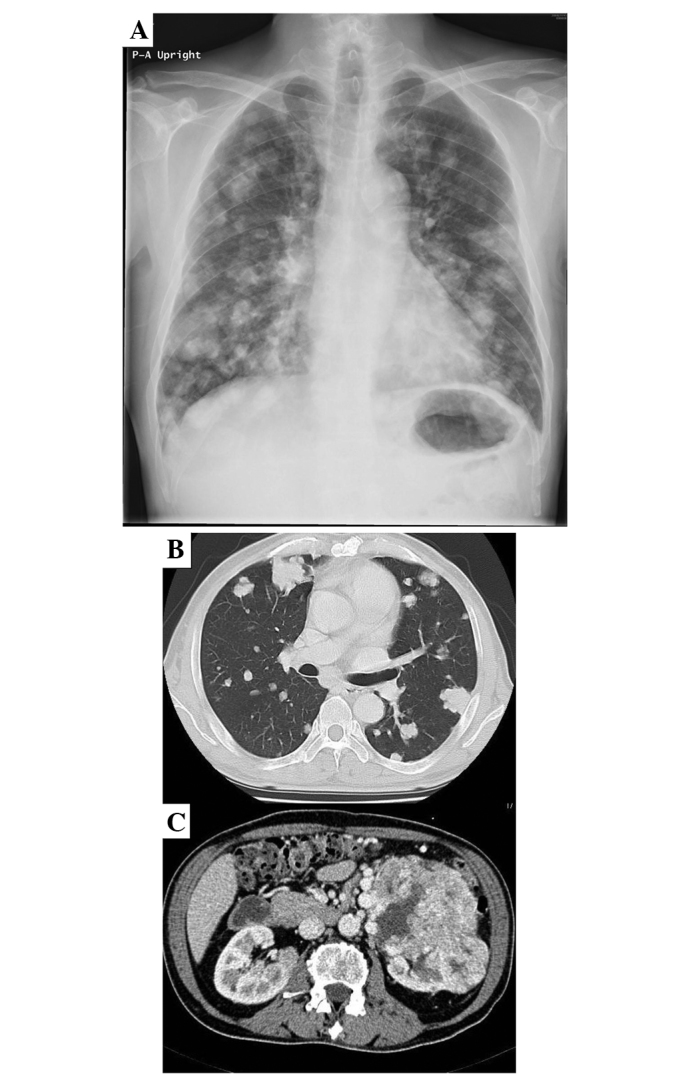
(A) Plain film demonstrating multiple metastatic lesions of the bilateral lungs. (B) Lung computed tomography (CT) also showing multiple metastatic lesions. (C) Screening of abdominal CT revealing left renal cell carcinoma.

A 59-year-old male was referred to the Department of Orthopedic Surgery, Osaka City University Hospital (Osaka, Japan)in February 2008. The patient presented with a seven-month history of gradually increasing pain in the right leg. The patient had previously visited Fuchu Hospital (Izumi, Japan) due to an abnormal shadow on the right proximal femur and was subsequently referred to our hospital. A plain film revealed an osteolytic lesion with an ill-defined margin in the right proximal femur (Fig. 1A), suggesting a malignant bone tumor. Pelvic CT also revealed a mass in the right ilium, extending into the gluteal muscle (Fig. 1B). Lung plain film (Fig. 2A) and CT (Fig. 2B) confirmed multiple masses in bilateral lung fields. Screening of the abdominal CT to detect the primary cancer revealed an occupying mass in the left kidney (Fig. 2C). Other metastases involving the pancreas and abdominal lymph nodes were also revealed. Subsequent to consultation with the Department of Urology and clinical staging, the patient was diagnosed with advanced-stage RCC (cT3aN3M1; stage IV). Resection of the primary RCC and palliative surgery with a γ-nail for an impending fracture of the right proximal femur were performed simultaneously, which revealed hemorrhagic brown tissue. The histology of a surgical specimen revealed that the tumor was composed of cells with clear cytoplasm and alveolar structural patterns. The pathological diagnosis of the surgical specimen of the curettage material was consistent with renal clear cell carcinoma.

At two weeks post-surgery, radiotherapy (36 Gy/12 fractions) was administered to the tumor in the right proximal femur for three weeks and subcutaneous injection of interferon-α (5×10^6^IU) was started (5 times per week, for 9 months). The patient then received 200 mg oral sorafenib combined with interferon-α every day for two weeks, subsequently the dosage of sorafenib was increased to 400 mg. No major adverse effects were experienced, but a dry skin rash developed on the face and trunk, and the patient experienced mild diarrhea. Subsequently, the dose of sorafenib was reduced to 200 mg for two weeks. Four weeks later, the dose was increased to 400 mg. Treatment with sorafenib was continued for eight months and the dose (400–600 mg) was determined according to the adverse effects experienced by the patient.

**Figure 3:**
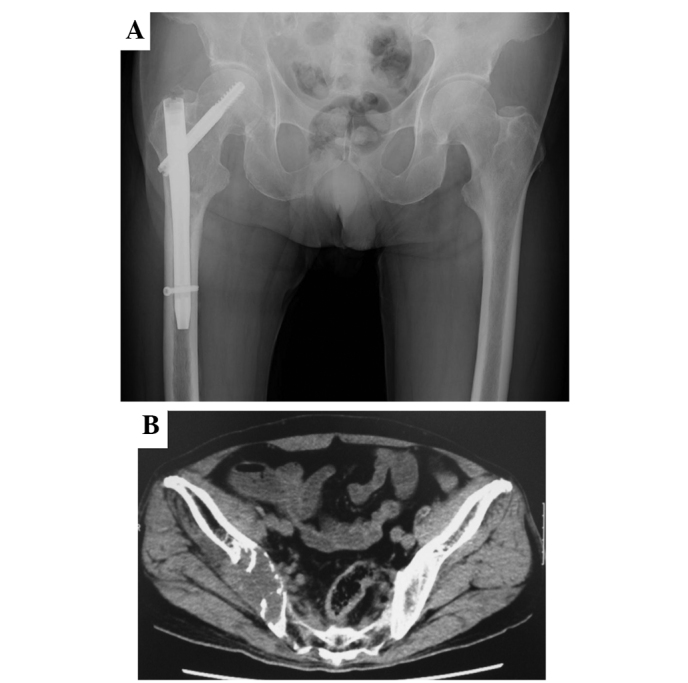
(A) Plain film at last eight months post-surgery showing the right femur with γ-nail fixation and no tumor progression. (B) Pelvic computed tomography confirming reduction of the mass in the ilium.

**Figure 4:**
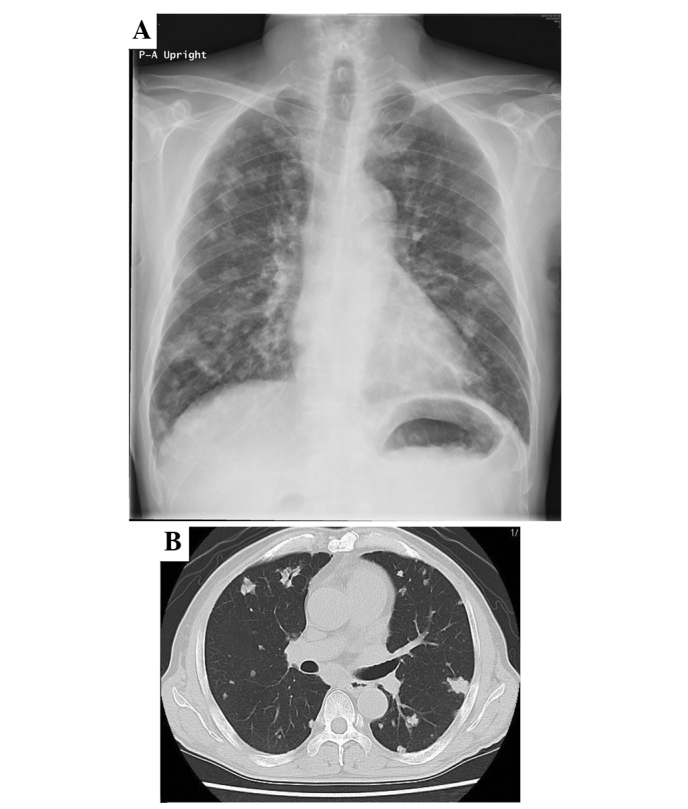
(A) Plain film and (B) CT of the lung at the last follow-up showing reduction in number and size of masses.

At eight months post-surgery, a plain film (Fig. 3A) showed no apparent progression in the right femur, and pelvic CT demonstrated regression of the mass in the right ilium (Fig. 3B). Plain film and CT of the lungs also revealed favorable responses (Fig. 4). The abdominal lymph node swelling was also reduced. The patient remained alive with the disease at the time of follow-up at 36 months post-surgery.

## Discussion

RCC is estimated to account for ~3.8% of all cancers (1). RCC has a high mortality rate, with a five-year survival rate of <10% (5), despite recent progress in various therapeutic strategies. The bone remains one of the most common distant metastatic sites of RCC. Patient quality of life is negatively impacted by bone damage caused by developing bone metastases (6), however, due to severe pain and a poor performance status, the administration of systemic chemotherapy is difficult. The prognosis is further worsened by the unfavorable conditions (7).

Interferon-α and interleukin-2 are generally accepted as the standard treatments for advanced-stage RCC (8,9). However, sorafenib, a multi-kinase inhibitor that blocks the raf and tyrosine kinases of vascular endothelial and platelet-derived growth factor receptors, has been recently introduced for the treatment of unresectable and/or multiple metastatic RCC (4). In a phase III randomized, placebo-controlled trial, sorafenib produced a response in 10% of patients who were resistant to standard therapy, and progression-free survival was significantly prolonged from 2.8 to 5.5 months (10). In a phase II study, progression-free survival in the sorafenib-treated group was not significantly different from that in the interferon-α-2a-treated group (10). In the current study, the patient developed minor adverse effects, including a skin rash on the face and trunk, and mild diarrhea, necessitating a temporary dose reduction. Major adverse effects (grade, >3) of hand-foot skin reactions (11.3%), diarrhea (6.2%) and rashes/desquamation (6.2%) have previously been reported (10).

There is little information available concerning the chemotherapeutic response of bone metastasis to sorafenib. Iliac bone metastases in the current patient were significantly reduced without the use of radiotherapy. The association between EGFR kinase inhibitors and bone metastases has rarely been investigated. Using prostate cancer cells, Angelucci *et al* (11) and Normanno and Gullick (12) suggested three possible pathways to explain the beneficial effects of EGFR inhibitors on bone metastases. Firstly, using cell invasion-related molecules, including matrix metalloproteinase-9 and urokinase-type plasminogen, EGFR tyrosine kinase inhibitors may act directly on tumor cells. Secondly, EGFR inhibitors may exert anti-angiogenic activity via the blockage of vascular endothelial growth factor production. Thirdly, EGFR tyrosine kinase inhibitors may cause the inhibition of osteoclast development by affecting the induction of osteoclast differentiation and activation by bone marrow stromal cells. These mechanisms could all act to prevent the expansion of bone metastases.

The one-year survival rate of patients after treatment for bone metastases from RCC was found to be 47%, when compared with 31.6% for lung cancer patients (13,14). However, the prognosis of the current patient at the initial presentation was considered to be poor due to the presence of multiple organ metastases, including lung, liver, pancreas and lymph node metastases. Orthopedic surgery was performed using γ-nail fixation as palliative therapy for preventing a pathological fracture of the right femur. However, molecularly targeted therapy with sorafenib led not only to bone recovery, but also improved the survival time.

In conclusion, the current study reports the case of a male patient with RCC who presented with bone metastases of the right proximal femur and ilium. Marked tumor reduction was observed subsequent to treatment with a combination of sorafenib and interferon-α for 9 months. The patient remained alive at the time of follow-up at 36 months post-surgery. Sorafenib administration is a promising agent for improving the prognosis of patients with bone metastases of RCC.
